# Recryopreservation impairs blastocyst implantation potential via activated endoplasmic reticulum stress pathway and induced apoptosis

**DOI:** 10.1002/mco2.689

**Published:** 2024-08-16

**Authors:** Meng Wang, Juepu Zhou, Rui Long, Yuehan Li, Limin Gao, Ruolin Mao, Xiangfei Wang, Na Guo, Lei Jin, Lixia Zhu

**Affiliations:** ^1^ Reproductive Medicine Center Tongji Hospital, Tongji Medical College, Huazhong University of Science and Technology Wuhan China

**Keywords:** blastocyst, endoplasmic reticulum stress, frozen embryo transfer, implantation, recryopreservation

## Abstract

Recryopreservation (recryo) is occasionally applied in clinical, while the underlying mechanism of impaired clinical outcomes after recryo remains unclear. In this study, frozen embryo transfer (FET) cycles of single blastocyst transfer in an academic reproductive medicine center were enrolled. According to the number of times blastocysts experienced cryopreservation, they were divided into the cryopreservation (Cryo) group and the Recryo group. Donated human blastocysts were collected and detected for mechanism exploration. It was found that recryo procedure resulted in impaired blastocyst developmental potential, including decreased implantation rate, reduced biochemical pregnancy rate, declined clinical pregnancy rate, higher early miscarriage rate, and lower live birth rate. Moreover, recryo led to impaired trophectoderm (TE) function, exhibiting lower human chorionic gonadotropin levels 12 days after FET. In addition, single‐cell RNA sequencing showed that the expression of genes involved in cell adhesion and embryo development were altered. More specifically, activated endoplasmic reticulum (ER) pathway and induced apoptosis were further verified by immunofluorescence and terminal deoxynucleotidyl transferase‐mediated dUTP nick‐end labeling (TUNEL) assay involving in the recryo procedure. In conclusion, recryo could interfere with the process of blastocyst implantation by impairing TE function, affecting blastocyst adhesion, activating ER stress pathway and inducing apoptosis. It provides caution to embryologists about the potential risk of recryopreservation.

## Graphical abstract

1. Recryo procedure in blastocysts results in impaired blastocyst developmental potential.

2. Recryo procedure impairs trophectoderm (TE) function of blastocysts.

3. Recryo procedure alters the transcriptome of human blastocysts.

4. Recryo procedure affects blastocyst adhesion, activates endoplasmic reticulum (ER) stress pathway and induces apoptosis

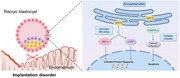

## 1 INTRODUCTION

Frozen embryo transfer (FET) is becoming increasingly prevalent owing to the advancement of assisted reproductive technology (ART) such as cryopreservation (cryo) technique. Various studies have illustrated the potential benefits of FET. Compared to fresh embryo transfer, FET lowers the prevalence of side effects of controlled ovarian hyperstimulation (COH).^1^, ^2^ and improves endometrial receptivity,^3^ resulting in a higher live birth rate.^4^ Moreover, the application of FET minimizes the risk of pregnancy and neonatal complications.^5^, ^6^ In addition, FET offers the advantage of allowing the application of preimplantation genetic testing (PGT) and fertility preservation.^7^

Because of the increasing utilization of FET, recryopreservation (recryo) is occasionally applied in several circumstances, such as multiple embryos in a single cryo‐straw, unexpected intubation failure, and rebiopsy in PGT cycles. Nevertheless, concerns about the effect of recryo procedure on clinical outcomes are rising. Several studies demonstrated comparable clinical pregnancy rates and live birth rates between cryo and recryo embryos,^8^ while conversely, some studies reported impaired implantation potential of embryos experiencing recryo procedure.^9^, ^10^ In a previous study, it was found that recryo procedure had an adverse effect on embryo implantation potential, exhibiting decreased implantation rate, reduced clinical pregnancy rate, lower live birth rate, as well increased miscarriage rate, albeit comparable neonatal outcomes,^11^ which was also reported in another study.^12^

Previous studies investigating recryo embryos were with a long time span, various cryopreservation methods and carriers, and different embryo stages of cryopreservation and warming, which may attribute to the variability and bias in results. As the high‐speed development of ART, vitrification is gradually becoming the leading cryopreservation method, and blastocyst transfer emerges as the main FET strategy, especially in PGT cycles.^7^ Therefore, exploring the effect of cryo and recryo on blastocysts possesses meaningful guidance for current field of ART. Recently, a meta‐analysis involving 14 studies suggested that recryo procedure impaired embryo viability and resulted in lower in vitro fertilization (IVF) success rates, without obviously damaging neonatal outcomes. Moreover, the subgroup analyses showed both implantation rate and live birth rate were notably affected in the recryo group, when comparing the embryos at blastocyst stages.^13^ However, the underlying mechanism of impaired clinical outcomes after recryo procedure remains unclear.

In this study, after controlling the potential confounding factors, including cryopreservation stage, cryopreservation method, carriers, biopsy disturbance and embryo quality, and expanding the sample size, we aim to investigate the effect of recryo procedure on blastocyst outcomes by using propensity score matching and explore the possible underlying mechanism by utilizing transcriptome sequencing.

## 2 RESULTS

### 2.1 Baseline characteristics comparison between the groups

**FIGURE 1:**
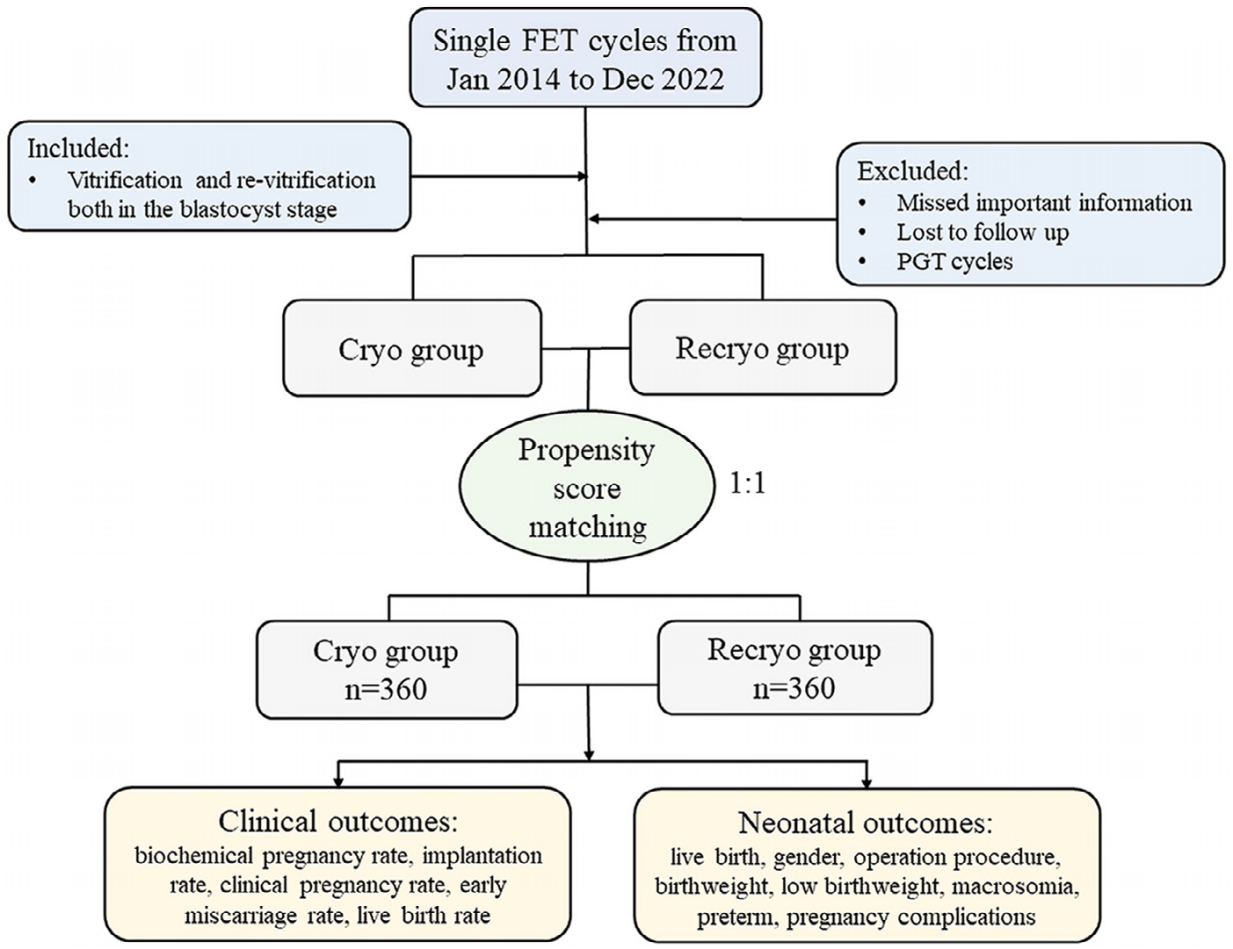
Flow chart of the clinical research. FET, frozen embryo transfer; PGT, preimplantation genetic testing.

In the comparison of the cryopreservation (Cryo) group and the recryopreservation (Recryo) group, there were 360 cycles in each group after propensity score matching (Figure 1), and the baseline characteristics between the two groups were similar (Table 1), in terms of age at oocyte retrieval, endometrial preparation protocol, endometrium thickness, blastocyst developmental stage, blastocyst grade, blastocyst expansion level, inner cellular mass (ICM) score, and trophectoderm (TE) score. The density of propensity scores of the two groups almost overlapped. (Figure S1A,B). Similarly, the distribution of standardized differences after matching was more concentrated (Figure S1C,D). Moreover, univariate standardized differences after matching basically concentrated around 0 (Figure S1E). In addition, individual differences after matching decreased significantly (Figure S1F). All the above results indicated the well‐balanced distribution between the comparing cohorts after matching.

**TABLE 1:** Baseline characteristics after matching.

	Cryo (*n* = 360)	Recryo (*n* = 360)	*p* value
Age at oocyte retrieval (year)	31 (28, 33)	31 (27, 35)	0.602
Endometrial preparation protocol			0.062
Hormone replacement treatment	95.6 (344)	92.2 (332)	
Natural cycle treatment	4.4 (16)	7.8 (28)	
Endometrium thickness (mm)	9.0 (8.3, 9.9)	9.0 (8.3, 9.7)	0.718
Developmental stage			0.954
D5	38.6 (139)	38.9 (140)	
D6	61.1 (220)	60.6 (218)	
D7	0.3 (1)	0.6 (2)	
Grade			0.873
Good	3.6 (13)	3.9 (14)	
Average	42.8 (154)	40.8 (147)	
Poor	51.7 (186)	53.9 (194)	
Others^a^	1.9 (7)	1.4 (5)	
Expansion level			0.866
1	0.0 (0)	0.3 (1)	
2	1.9 (7)	1.1 (4)	
3	28.1 (101)	30.0 (108)	
4	59.7 (215)	59.2 (213)	
5	5.0 (18)	5.0 (18)	
6	5.3 (19)	4.4 (16)	
ICM score^b^			0.869
A	2.8 (10)	3.1 (11)	
B	93.9 (338)	93.6 (337)	
C	1.4 (5)	1.9 (7)	
Others	1.9 (7)	1.4 (5)	
TE score^b^			0.926
A	2.8 (10)	2.5 (9)	
B	44.7 (161)	44.2 (159)	
C	50.6 (182)	51.9 (187)	
Others	1.9 (7)	1.4 (5)	

Abbreviations: ICM, inner cellular mass; TE, trophectoderm.

^a^ Blastocysts with “others” grade referred to those with expansion level of 1 and 2 based on the Gardner scoring system.

^b^ ICM and TE scores were based on the Gardner scoring system. Blastocysts with “others” of ICM or TE score referred to those with expansion level of 1 and 2.

### 2.2 Recryo procedure in blastocysts results in impaired blastocyst developmental potential

Clinical outcomes of the FET cycles between the two groups after matching were presented in Table 2. Compared to once cryo, recryo procedure in blastocysts resulted in impaired blastocyst developmental potential, including decreased implantation rate (40.0% vs. 47.8%, *p *= 0.035), reduced biochemical pregnancy rate (45.3% vs. 58.6%, *p *< 0.001), declined clinical pregnancy rate (40.0% vs. 47.8%, *p *= 0.035), higher early miscarriage rate (31.3% vs. 20.3%, *p *= 0.026), and lower live birth rate (24.7% vs. 34.4%, *p *= 0.004). However, there were no significant differences regarding the neonatal outcomes of the transferred blastocysts after matching (Table 3). The gender, birthweight, pregnancy complications, and neonatal complications were similar between the groups, despite a higher proportion of cesarean sections in the Recryo group (93.3% vs. 83.1%, *p *= 0.027).

**TABLE 2:** Clinical outcomes after matching.

	Cryo (*n* = 360)	Recryo (*n* = 360)	OR 95% CI	*p* value
Biochemical pregnancy rate	58.6 (211/360)	45.3 (163/360)	1.71 (1.27, 2.30)	<0.001
Implantation rate	47.8 (172/360)	40.0 (144/360)	1.37 (1.02, 1.84)	0.035
Clinical pregnancy rate	47.8 (172/360)	40.0 (144/360)	1.37 (1.02, 1.84)	0.035
Early miscarriage rate	20.3 (35/172)	31.3 (45/144)	0.56 (0.34, 0.94)	0.026
Live birth rate	34.4 (124/360)	24.7 (89/360)	1.60 (1.16. 2.21)	0.004

Abbreviations: CI, confidence interval; OR, odds ratio.

**TABLE 3:** Neonatal outcomes after matching.

	Cryo	Recryo	OR 95% CI	*p* value
Live birth	124	89		
Gender			0.84 (0.49, 1.45)	0.524
Male	48.4 (60)	52.8 (47)		
Female	51.6 (64)	47.2 (42)		
Operation procedure			0.35 (0.14, 0.92)	0.027
Cesarean section	83.1 (103)	93.3 (83)		
Spontaneous labor	16.9 (21)	6.7 (6)		
Birthweight (kg)	3.3 (3.1, 3.6)	3.4 (3.1, 3.8)		0.698
Low birthweight^a^	8.1 (10)	5.6 (5)	1.47 (0.49, 4.47)	0.491
Macrosomia^b^	9.7 (12)	7.9 (7)	1.26 (0.47, 3.33)	0.647
Preterm^c^	13.7 (17)	16.9 (15)	0.78 (0.37, 1.67)	0.526
Pregnancy complications	8.1 (10)	15.7 (14)	0.47 (0.20, 1.11)	0.081

Abbreviations: CI, confidence interval; OR, odds ratio.

^a^ Low birthweight: birth weight <2500 g.

^b^ Macrosomia: birth weight >4000 g.

^c^ Preterm: gestational week <37 weeks.

### 2.3 Recryo procedure impairs TE function of blastocysts

**FIGURE 2:**
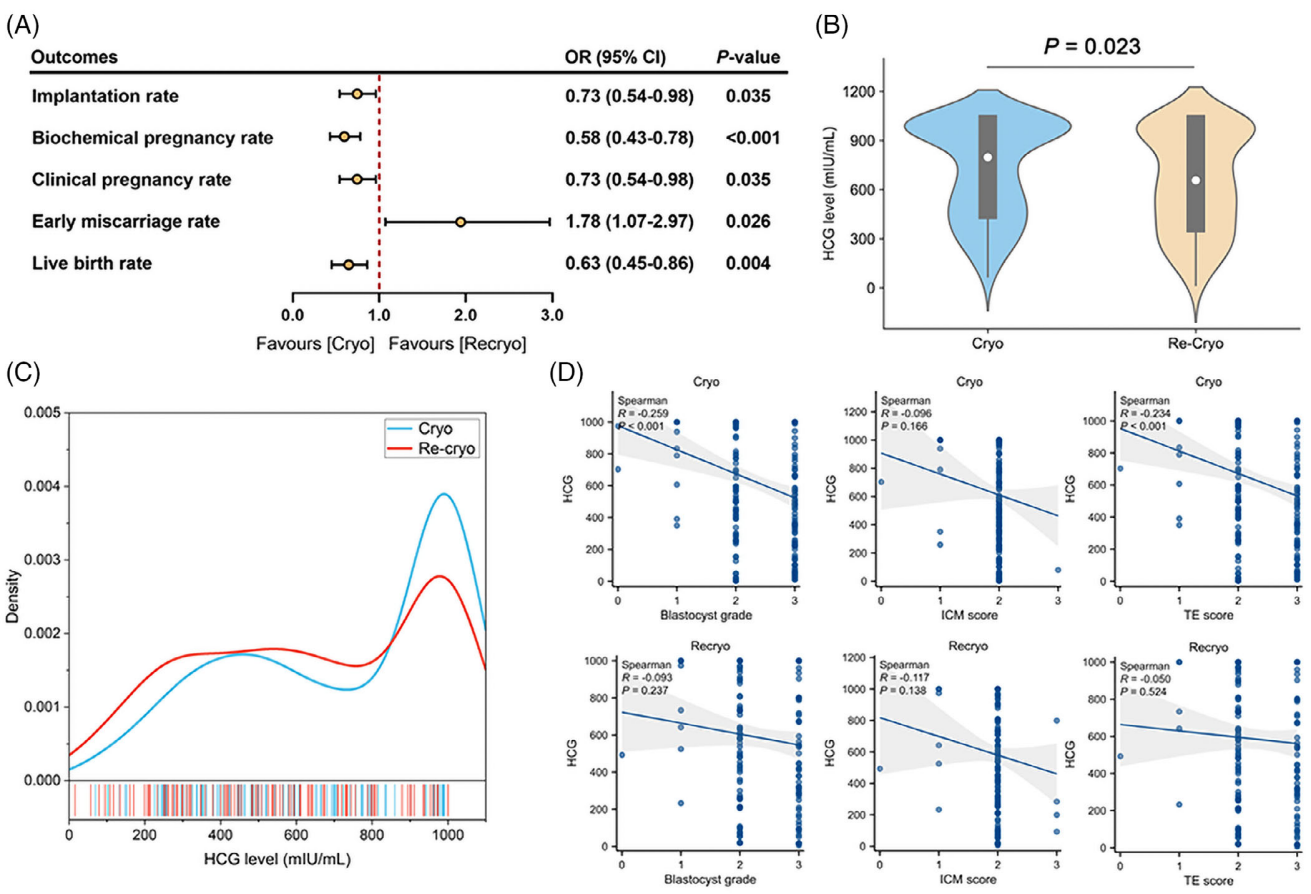
Recryo impairs trophectoderm (TE) function of blastocysts. (A) Clinical outcomes comparison between recryo blastocysts and cryo blastocysts after matching. (B) Violin plot of human chorionic gonadotropin (HCG) level 12 days after blastocyst transfer. (C) Density plot of HCG level 12 days after blastocyst transfer. (D) Correlation plot of blastocyst grade, inner cellular mass (ICM) score, and TE score with HCG level 12 days after blastocyst transfer.

As Figure 2A shown, blastocysts experiencing recryo procedure had impaired developmental potential, including a decreased implantation rate. Since human chorionic gonadotropin (HCG) level 12 days after blastocyst transfer is associated with implantation competence of transferred blastocysts, the HCG levels between the two groups were compared. There were 172 blastocysts implanted in the Cryo group and 144 in the Recryo group. It was shown that the median HCG level was lower in the Recryo group (657 [385–1000] vs. 798 [469–1000], *p *= 0.023, Figure 2B), and the distribution of HCG level in the Cryo group was more concentrated around the detected upper limit (Figure 2C). To explore the association between HCG level and blastocyst grade, correlation analyses were performed. In Figure 2D, it was found that HCG level was positively correlated with TE quality (*p *= 0.002) and it increased with a higher blastocyst grade (*p *< 0.001). While interestingly, after recryo procedure, the correlation between HCG level and blastocyst grade showed no significance, so did the association with TE score. These results prompted that recryo procedure impairs TE function of blastocysts and HCG secretion.

### 2.4 Recryo procedure alters the transcriptome of human blastocysts

**FIGURE 3:**
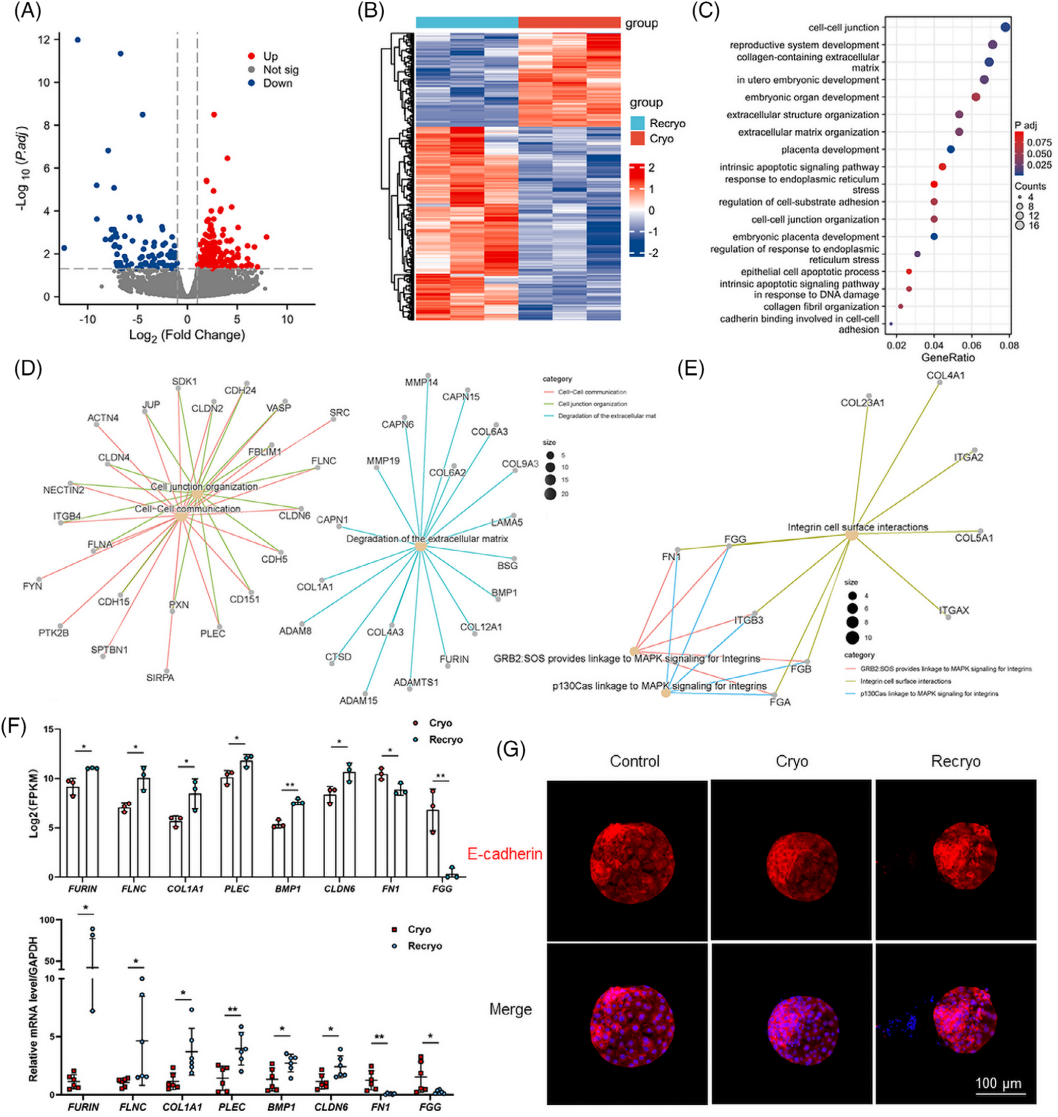
Recryo alters gene expression involving in embryo implantation in human blastocysts. (A) Volcano plot of differentially expressed genes (DEGs) in blastocysts between Recryo and Cryo groups. (B) Heatmap of DEGs in blastocysts between Recryo and Cryo groups. (C) Gene ontology (GO) analyses of DEGs in blastocysts between Recryo and Cryo groups. (D) The upregulated DEGs associated with embryo implantation were involved in cell–cell communication/cell junction organization and degradation of the extracellular matrix. (E) The downregulated DEGs were related with integrin cell surface interactions. (F) Real‐time polymerase chain reaction (RT‐PCR) was performed to validate the accuracy of the RNA sequencing. (G) Immunofluorescence staining of E‐cadherin in blastocysts from different groups.

To explore the effect of recryo procedure on the gene expression characteristics of human blastocysts, single‐cell RNA sequencing was performed in recryo and cryo blastocysts. There were 291 differentially expressed genes (DEGs) with 196 upregulated and 95 downregulated (Figure 3A,B). Gene ontology (GO) analyses showed that the DEGs mainly participated in the process of cell adhesion, embryo development, and apoptosis (Figure 3C). To be more specific, the upregulated DEGs associated with embryo implantation were involved in cell–cell communication/cell junction organization (*SDK1*, *SRC*, *FLNC*, *CLDN6*, *CDH5*, *PLEC*, and *CLDN4*) and degradation of the extracellular matrix (*COL1A1*, *CTSD*, *COL4A3*, *ADAMTS1*, *FURIN*, *COL12A1*, and *BMP1*; Figure 3D). Moreover, the downregulated DEGs were related to Integrin cell surface interactions (*FN1* and *FGG*; Figure 3E). Several abovementioned genes exhibiting significantly different expression, including *FURIN*, *FLNC*, *COL1A1*, *PLEC*, *BMP1*, *CLDN6*, *FN1*, and *FGG*, were further confirmed by quantitative real‐time polymerase chain reaction (RT‐PCR), and the mRNA expression was in accordance with the results of RNA sequencing, confirming the accuracy of the RNA sequencing (Figure 3F). In addition, the localization of adhesion‐related E‐cadherin was detected (Figure 3G). E‐cadherin was evenly distributed on the cell membrane and arranged neatly, clearly, and continuously in fresh blastocysts in the control group. In the Cryo group, the arrangement of E‐cadherin is slightly disordered, unclear, and partially discontinuous. In the Recryo group, the E‐cadherin arrangement was chaotic and disorganized, with no distribution on the surface of some cells.

### 2.5 Recryo procedure activates endoplasmic reticulum stress pathway and induces apoptosis

**FIGURE 4:**
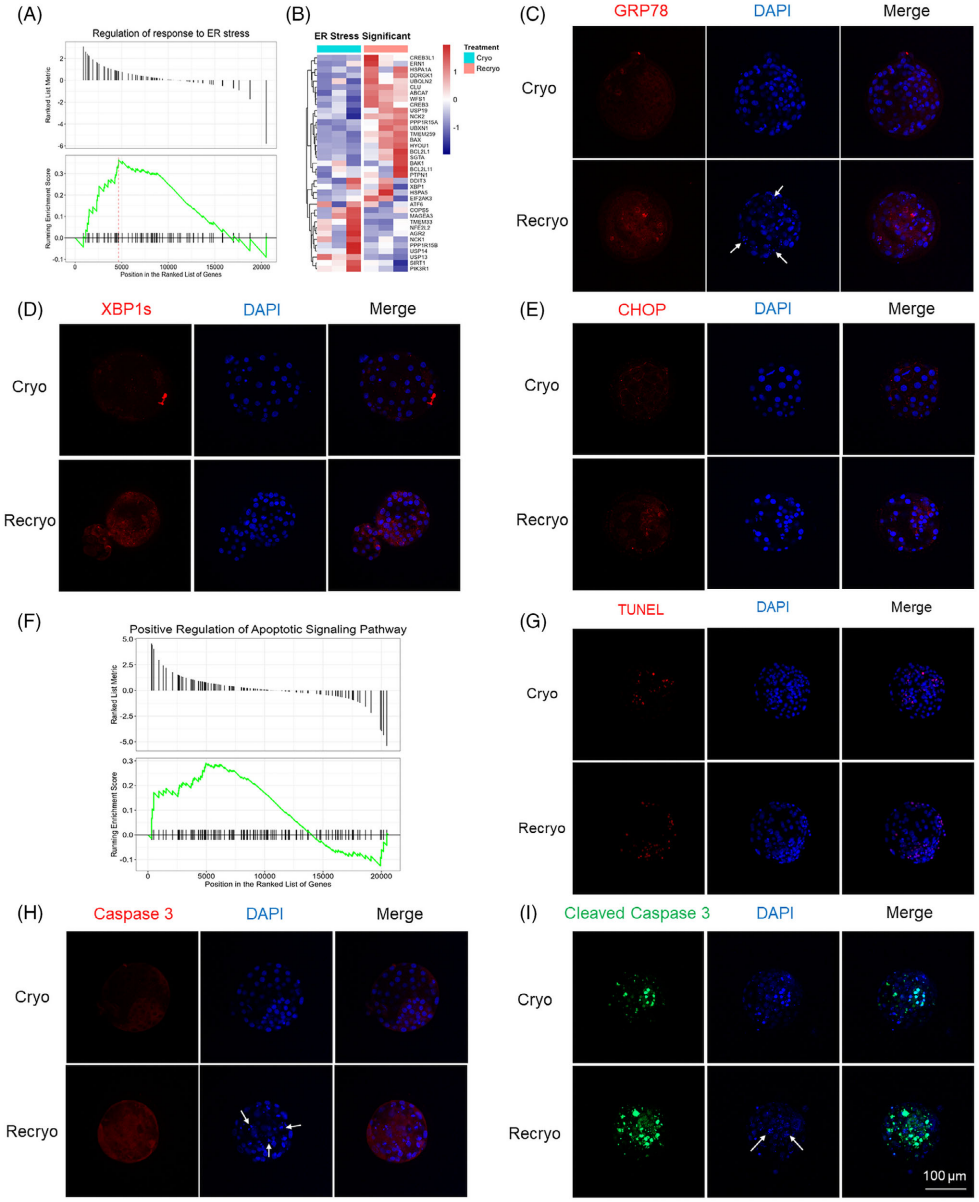
Recryo activates endoplasmic reticulum stress and induces apoptosis in human blastocysts. (A) Gene set enrichment analysis (GSEA) showed activated regulation of response to endoplasmic reticulum (ER) stress after recryo. (B) Heatmap of significant genes involved in ER stress. (C) Protein expression of GRP78 between the groups. (D) Protein expression of XBP1s from the Cryo and Recryo groups. (E) Protein expression of CHOP from different groups. (F) GSEA analysis showed the positive regulation of apoptotic signaling pathway after recryo. (G) Terminal deoxynucleotidyl transferase‐mediated dUTP nick‐end labeling (TUNEL) staining of blastocysts in Cryo and Recryo groups. (H) Protein expression of Caspase‐3 from different groups. (I) Detection of cleaved Caspase‐3 in blastocysts.

GSEA analysis showed that the regulation of response to endoplasmic reticulum (ER) stress was activated after recryo (Figure 4A). In Figure 4B, altered genes in ER stress were visualized by heatmap. The expression of several important proteins involved in ER stress, including GRP78, XBP1s, and CHOP, was detected by immunofluorescence staining (Figure 4C–E). After the recryo procedure, the expression of GRP78 increased, which activated the downstream protein expression of XBP1s and CHOP. In addition, it was found that there was a positive regulation of the apoptotic signaling pathway (Figure 4F). Compared with blastocysts in the Cryo group, the apoptotic cells in the Recryo group were significantly increased by terminal deoxynucleotidyl transferase‐mediated dUTP nick‐end labeling (TUNEL) assay (Figure 4G). Moreover, the expression of both Caspase‐3 (Figure 4H) and cleaved Caspase‐3 (Figure 4I) was upregulated after recryo, indicating the activation of apoptosis‐associated caspase cascade reaction. In addition, the number of fragmented nuclei, which were indicated by white arrows, was significantly increased in blastocysts from the Recryo group.

## 3 DISCUSSION

In the current study, we found that recryo procedure in blastocysts resulted in impaired blastocyst developmental potential, including decreased implantation rate, reduced biochemical pregnancy rate, declined clinical pregnancy rate, higher early miscarriage rate, and lower live birth rate, albeit similar neonatal outcomes. Moreover, recryo led to impaired TE function, exhibiting lower HCG levels 12 days after FET. In addition, single‐cell RNA sequencing showed that the expression of genes involved in cell adhesion, embryo development, ER stress pathway, and apoptosis were altered, which were further confirmed by immunofluorescence and TUNEL assay. Our data showed that recryo procedure could interfere with the process of implantation by impairing TE function, affecting blastocyst adhesion, activating ER stress pathway, and inducing apoptosis.

Currently, there is continuing debate as to whether recryo procedure in embryos affects the clinical outcomes. Studies investigating the effect of recryo procedure on embryo clinical outcomes reported contradictory data owing to the variety of sample sizes, study designs, and inclusion criteria. Several studies demonstrated similar clinical pregnancy outcomes and live birth rates regardless of the times of embryo experiencing cryo procedures. While conversely, some other studies reported impaired implantation potential and subsequently reduced live birth rate and increased miscarriage rate. In addition, some research suggested that recryo procedure can affect several neonatal outcomes. In our study, we enrolled 360 cycles after matching in each group, with 124 live birth in the Cryo group and 89 in the Recryo group, and the current study was with the largest sample size ever reported. It should be noted that the variability of results of previous studies may be attributed to various confounding factors, while our study controlled them as possible, including cryopreservation stage, cryopreservation method, carriers, biopsy disturbance, embryo quality and thus only the effect of recryo procedure on embryos was investigated. Our findings showed that recryo procedure caused decreased implantation rate, reduced live birth rate, and increased miscarriage rate, which corroborated with a recently published meta‐analysis enrolling 13 cohort studies and one case–control study, in which the authors highlighted a lower rate of IVF success after repeated cryopreservation.^13^ In summary, the current study reinforced that embryologists should remain a cautious attitude toward the utilization of embryos experiencing repeated cryopreservation.

The process of implantation is the interaction between TE cells and endometrial cells, including sequential phases of apposition, attachment, and invasion. Apposition involves weak contact with the endometrium through TE cells before more strengthened molecular adhesions, such as cadherins, integrins, and selectins, during the attachment process. At the invasion stage, the TE cells proliferate and differentiate into specialized subtypes of syncytiotrophoblast and cytotrophoblast to help with invasion into the stromal decidual tissue.^14^ In addition, previous studies have demonstrated that TE grade is significantly correlated with implantation and live birth.^15^ Thus, maintaining the normal function of TE cells ensures the success of implantation and subsequent fetus development. β‐hCG is a hormone secreted by placental syncytiotrophoblast tissue during early pregnancy, and the measurements and monitoring of serum β‐hCG concentration are utilized for pregnancy confirmation, as well as the differential diagnosis of normal pregnancy and others. It was reported that serum hCG levels were predictive of embryo implantation potential,^16^ and it has been demonstrated that β‐hCG kinetics differed regarding blastocysts quality and clinical outcomes.^17^ Moreover, it has been theorized that TE grades correlated with β‐hCG levels at first measurement.^18^ Therefore, the hCG level is reflective of embryo TE grade and might be critical for embryo–endometrium crosstalk during implantation. Some other studies proposed that cryopreservation may impair the secretion of β‐hCG.^19^ In this study, we found that hCG levels in the recryo group were lower, indicating impaired TE function and implantation potential of recryo blastocysts, which was consistent with the declined implantation rate observed in the recryo group in our study. Furthermore, a correlation between TE grade with β‐hCG measurement was investigated, and it was found that the presence of recryo procedure interfered with the positive linear relationship between TE grade and hCG level, suggesting that recryo procedure might act as a negative confounding factor for TE function and implantation.

Many studies have demonstrated that vitrification altered transcriptional profiles of preimplantation embryos, including genes involved in apoptosis (*BCL2*, *TP53*, *BAX*, and *BCL2L1*), stress responses (*HSPA5*), pluripotency (*POU5F1*, *NANOG*, and *SOX2*), zygotic genome activation (*EIF1AX* and FIGLA), cell differentiation (*KRT19* and *CLDN23*), and implantation (*PTGS2* and *CALB1*).^20^, ^21^, ^22^, ^23^, ^24^ However, few studies have ever focused on the effect of repeated cryopreservation on the embryo genome. In an experimental study using the mouse to explore the effect of recryo, it was found that recryo mouse blastocysts showed higher expression levels of apoptotic genes (*BAX* and *CASP3*),^25^ which was in line with our study. In the current study, we found that after recryo, altered genes were involved in the process of cell adhesion, embryo development, and apoptosis. In addition, animal evidence showed that the DNA integrity can be affected by vitrification of blastocysts,^26^ and so did our study, in which the DNA integrity of human blastocysts were assessed by TUNEL and the protein expression of Caspase‐3 and cleaved Caspase‐3 were also detected. More specifically, in our study it was found that ER stress, which plays a vital role in cell proliferation and survival,^27^, ^28^ was involved in this process of recryo, exhibiting elevated expression of GRP78, XBP1s, and CHOP. Therefore, we postulated that the recryo process may impair cell adhesion, activate ER stress, lead to apoptosis, and finally result in implantation failure. More in‐depth mechanism explorations are needed to explain our clinical investigations.

To date, the number of studies investigating the neonatal outcome resulting from recryo procedure was limited. Although our findings together with several other studies suggested that recryo procedures have no clear impact on neonatal outcomes,^29^ the utilization of recryo embryos still requires careful consideration for safety reasons. Additional operations, such as laser drilling, might harm embryo developmental potential. The possible DNA damage or congenital disorders secondary to the application of artificial laser has gained great concerns.^30^ In addition, prolonged in vitro culture time before ET increased the risk of adverse cardiovascular events in offspring in mice,^31^ challenging the long‐term safety of the consumption of recryo embryos. However, there is comparatively little information in the published research involving embryos of both animals and humans that provide clues about the safety of recryo application on embryos. Thus, more robust evidence of long‐term offspring health with more participants is needed to support the safety of the recryo procedure.

Our study reported the largest sample size of recryo blastocysts ever, and it was the first study exploring the potential molecular mechanism of the effect of recryo on human blastocysts. However, there were still several limitations. The nature of the retrospective cohort study limited the evidence grade of our results, despite the use of propensity score matching to minimize potential selection bias. Moreover, in the Recryo group, some patients have experienced several cycles of implantation failure, which can inevitably lead to bias. In addition, only blastocysts that were frozen and refrozen at the blastocyst stage were enrolled in our study, which cannot represent all recryo embryos, since different developmental stages of embryos during cryopreservation may lead to different results.

In conclusion, our findings demonstrated that recryo procedure in blastocysts impaired embryo implantation potential with lower implantation rate and live birth rate, which may be attributed to the altered molecular levels of genes involving in activated ER stress pathway and induced apoptosis after recryo, followed by abolished cell adhesion and decreased embryo development viability. Although the application of recryo procedure avoids the wastage of embryos and facilitates emergency handling, it provides caution to embryologists about the potential risk of recryopreservation on embryos. Moreover, the possible long‐term consequences of offspring health warrant further studies with a larger sample size.

## 4 MATERIALS AND METHODS

### 4.1 Study design and ethical approval

In this retrospective study, we enrolled all FET cycles of single blastocyst transfer between January 2014 and December 2022 in the Reproductive Medicine Center of Tongji Hospital. According to the number of times blastocysts experienced cryopreservation, the FET cycles were divided into the Cryo group and the Recryo group. In the Cryo group, embryos were vitrified in the blastocyst stage, then cultured and transferred. In the Recryo group, the vitrification and revitrification procedures were both performed in the blastocyst stage. Couples who underwent PGT were excluded. The indications of recryo procedure and recryo blastocysts consumption were well described as previously reported.^11^ The baseline characteristics, clinical outcomes, and neonatal outcomes were compared between the Cryo and Recryo groups.

Furthermore, in order to elucidate the molecular mechanisms of the effect of recryo on embryo potential, human embryos were collected and detected. All the embryos investigated in this research were donated by the patients with written informed consent. Fresh blastocysts were from discarded polypronuclei originated embryos. Cryo blastocysts were donated from successful delivery patients, and recryo blastocysts were available from warmed cryo blastocysts experiencing repeated vitrification and warm. This study was approved by the Ethical Committee of Tongji Hospital (TJ‐IRB20211280).

### 4.2 Controlled ovarian stimulation and embryo culture

Controlled ovarian stimulation was performed based on standard gonadotropin‐releasing hormone agonist or antagonist protocols.^32^ Oocytes were retrieved under the guidance of transvaginal ultrasound 36–38 h after HCG administration. The retrieved oocytes were fertilized in either conventional IVF or intracytoplasmic sperm injection (ICSI) when appropriate. The presence of 2 pronuclei (2PN) indicated successful fertilization after insemination. The embryos were cultured in G1‐plus medium (Vitrolife) until Day 3 and then cultured in G2‐plus medium (Vitrolife) to the blastocyst stage at 37°C with 5% O_2_ and 6% CO_2_. The blastocyst grade was based on the Gardner scoring system, and the expansion level, ICM score, and TE score were recorded. The morphology assessment was conducted by two experienced embryologists independently, with a third consulted in cases of inconsistency.

### 4.3 Vitrification and warming procedures

A commercial vitrification kit (Kitazato) was used for blastocyst cryopreservation as previously described.^33^ Artificial shrinkage using a laser was performed before vitrification. Blastocysts were transferred into equilibration solution (ES) for 5–8 min and then infiltrated into vitrification solution (VS) for 40–60 s. The blastocysts were immediately loaded on the tip of the Cryotop (Kitazato) and submerged in liquid nitrogen. For blastocyst warming, the Cryotop was immersed in preheated thawing solution (TS) (Kitazato) for 1 min. The blastocysts were then transferred to dilution solution (DS) for 3 min, 5 min in washing solution 1 (WS1) (Kitazato), and another 5 min in washing solution 2 (WS2). After 2 h culture, blastocyst viability was assessed and survival blastocysts were transferred.

### 4.4 Laboratory and clinical outcomes

Blastocyst transfer was conducted after natural cycle or hormone replacement treatment in our IVF center. Twelve days later, serum HCG was measured using the direct chemiluminescence ADVIA Centaur ThCG kit (Cat:00643953, Siemens). A positive result of HCG measurement was regarded as biochemical pregnancy. The presence of intrauterine gestational sac with active fetal heart using ultrasound 5 weeks after FET was defined as clinical pregnancy. Pregnancy loss within 20 weeks was regarded as early miscarriage. Birth weight lower than 2500 g was defined as low birthweight, and those above 4000 g were defined as macrosomia. Pregnancy complications included several diseases, including gestational diabetes mellitus, hypertensive disorders complicating pregnancy, placenta previa, and placenta abruption. The details of the computing formula of the laboratory and clinical outcomes can be found in previous studies.^34^

### 4.5 RNA sequencing and bioinformatics analysis

To investigate the effect of recryo procedure on blastocyst transcriptome, RNA sequencing was performed on cryo and recryo human blastocysts. To eliminate the effect of different sample sources on RNA transcriptome, three vitrified blastocysts (Cryo group) and three revitrified blastocysts (Recryo group) from the same couple were collected for RNA sequencing. The raw data have been deposited in the Genome Sequence Archive (GSA‐Human: HRA007913).^35^, ^36^ The warming blastocysts were cultured in G2‐plus medium for another day to partial or complete hatching (expansion level of 5 or 6) and then were collected for RNA sequencing. The commercial Smart‐Seq2 based on an Illumina NovaSeq 6000 platform was performed as previously reported by Annoroad Gene Technology. The DEGs between the two groups were analyzed using DESeq2 in R. Genes with adjusted *p* value <0.05 and absolute log2 (fold changes) >1.5 were considered statistically significant. DEGs expression was visualized using volcano plot and heatmap by the “ggplot2” package. GO analysis and enriched pathway analysis for DEGs were conducted by the “clusterProliler” package. In addition, gene set enrichment analysis (GSEA) was also performed using the “clusterProliler” package.

### 4.6 Quantitative real‐time polymerase chain reaction

RT‐PCR was performed to validate the results of RNA sequencing. Cryo and recryo blastocysts were warmed and cultured for another day and then four to five blastocysts were collected in one tube. Total RNA extraction and amplification were performed using Single Cell Sequence Specific Amplification Kit (Cat: P621‐01, Vazyme). Quantitative RT‐PCR was performed using ChamQ Universal SYBR qPCR Master Mix (Cat: Q711‐02, Vazyme) on the LightCycler 480 system (Roche). The relative mRNA expression normalized to *GAPDH* expression. The primer sequences are listed in Table S1.

### 4.7 Immunofluorescence and TUNEL assay

The immunofluorescence staining procedure was previously described and performed on fresh, cryo, and recryo blastocysts.^37^, ^38^ Fully expanded blastocysts after warming were fixed in 4% paraformaldehyde. Anti‐E‐cadherin antibody (Cat: A20798) was purchased from Abclonal, and anti‐Caspase‐3 (Cat: 19677‐1‐AP) was from Proteintech. Anti‐GRP78 (Cat: C50B12), anti‐XBP1s (Cat: 83418S), and anti‐CHOP (Cat: L63F7) antibodies were from Cell Signaling Technology. Primary antibodies were used at 1:100 dilution unless otherwise indicated, and Cy3 conjugated secondary antibodies (Cat: GB21301 for anti‐mouse and GB21303 for anti‐rabbit, Servicebio) were used at 1:100 dilution. The nuclei were stained with 4’,6‐diamidino‐2‐phenylindole (DAPI, Cat: G1012, Servicebio). Caspase‐3 activity detection kit for live cell (Cat: C1073M, Beyotime) was utilized for visualization of cleaved Caspase‐3. The TUNEL (Cat:C1089, Beyotime) assay was performed for apoptosis detection according to the manufacturer's instructions. Images were captured using a confocal microscope (LSM900, Leica).

### 4.8 Statistical analysis

Statistical analysis in the current study was performed using SPSS Statistics software (v26.0, IBM). Continuous variables are shown as medians (first quartile, third quartile), the comparison of which was achieved using Mann–Whitney *U* rank‐sum test. The categorical variables are shown as % (*n*/*N*), which were compared with chi‐squared test or Fisher's exact test as appropriate. A *p* value less than 0.05 (two‐tailed) was considered of statistical significance.

For the convenience of statistics, the “1,” “2,” and “3” scores of ICM or TE corresponded to A, B, and C in the Gardner scoring system. The transferrable blastocysts were divided into three groups based on the ICM and TE scores: good (AA, AB, and BA), average (BB), poor (AC and BC), and others (blastocysts with expansion levels of 1 or 2). Considering that several blastocysts were with expansion levels of 1 or 2, “4” score of ICM or TE was assigned to these blastocysts.

When comparing the two groups, to eliminate the imbalance of the sample size between the Cryo group and the Recryo group, propensity score matching was performed. The demographic characteristics were matched in a 1:1 ratio, including age at oocyte retrieval (year), endometrial preparation protocol (hormone replacement treatment and nature cycle treatment), endometrium thickness on the day of progesterone administration (mm), blastocyst developmental stage (D5, D6, and D7), blastocyst grade (good, average, poor, and others), blastocyst expansion level (1, 2, 3, 4, 5, and 6), ICM score (1, 2, 3, and 4), and TE score (1, 2, 3, and 4). Nearest neighbor matching strategy in random order without replacement (caliper = 0.1) was performed.

Owing to the detection limitation of the kit, the exact HCG values that were higher than 1000 mIU/mL will not be reported, and these values were set as 1000 when analyzing. The distribution of HCG level at 12 days after FET between the groups was visualized as violin plot and density plot using R. Correlation analysis of HCG level and blastocyst grade was performed using ggplot2 in R.

## AUTHOR CONTRIBUTIONS

*Designing research studies*: Lixia Zhu and Lei Jin. *Conducting experiments*: Meng Wang, Juepu Zhou and Rui Long. *Acquiring data*: Ruolin Mao. *Analyzing data*: Lixia Zhu, Meng Wang, Juepu Zhou and Ruolin Mao. *Sample collecting*: Lixia Zhu, Meng Wang, Juepu Zhou, Na Guo, Xiangfei Wang, Limin Gao and Yuehan Li. *Writing the manuscript*: Meng Wang, Juepu Zhou and Lixia Zhu. *Funding*: Lei Jin and Lixia Zhu. Lixia Zhu and Lei Jin were both corresponding authors. All authors have read and approved the final manuscript.

## CONFLICT OF INTEREST STATEMENT

The authors declare no conflicts of interest.

## ETHICS STATEMENT

This study was approved by the Ethical Committee of Tongji Hospital (TJ‐IRB20211280).

## Supporting information

Supporting Information

## Data Availability

The raw sequence data reported in this paper have been deposited in the Genome Sequence Archive in National Genomics Data Center, China National Center for Bioinformation/Beijing Institute of Genomics, Chinese Academy of Sciences (GSA‐Human: HRA007913) that are publicly accessible at https://ngdc.cncb.ac.cn/gsa‐human
