# Supplementary material for: Recryopreservation impairs blastocyst implantation potential via activated endoplasmic reticulum stress pathway and induced apoptosis
Source: MedComm (2020). 2024 Aug 16;5(9):e689. doi: 10.1002/mco2.689 (PMC11329749; doi:10.1002/mco2.689)

**Supplementary Information for**

**Re-cryopreservation impairs blastocyst implantation potential via activated endoplasmic reticulum stress pathway and induced apoptosis**

**Running title:** Re-cryopreservation impairs blastocyst implantation

Meng Wang, M.D.^1^; Juepu Zhou, M.D.^1^; Rui Long, M.D.^1^; Yuehan Li, M.D.^1^; Limin Gao, M.D.^1^; Ruolin Mao, M.M.^1^; Xiangfei Wang, M.D.^1^; Na Guo, M.D.^1^; Lei Jin, M.D., PhD.^1^*; Lixia Zhu, M.D., PhD.^1^*

^1^ Reproductive Medicine Center, Tongji Hospital, Tongji Medical College, Huazhong University of Science and Technology, Wuhan, China

**Corresponding authors:**

Lixia Zhu (zhulixia027@163.com) and Lei Jin (leijintjh@163.com)

Reproductive Medicine Center, Tongji Hospital, Tongji Medical College, Huazhong University of Science and Technology, Wuhan, 430030, China. Tel: (+86) 027-83662534; Fax: (+86) 027-83662534.

**Table S1 Primer sequences for RT-PCR**

| **Gene** | **F (5’-3’)** | **R (5’-3’)** |
| --- | --- | --- |
| *FURIN* | CCTGGTTGCTATGGGTGGTAG | AAGTGGTAATAGTCCCCGAAGA |
| *FLNC* | CTGGGCGATGAGACAGACG | GCGGATGGAACTTGCGGTA |
| *COL1A1* | GAGGGCCAAGACGAAGACATC | CAGATCACGTCATCGCACAAC |
| *PLEC* | CGATGCGACAACTTCACCTC | GCCGGTACACCTTGTTCATGT |
| *BMP1* | GGGTCATCCCCTTTGTCATTG | GCAAGGTCGATAGGTGAACACA |
| *CLDN6* | TGTTCGGCTTGCTGGTCTAC | CGGGGATTAGCGTCAGGAC |
| *FN1* | CGGTGGCTGTCAGTCAAAG | AAACCTCGGCTTCCTCCATAA |
| *FGG* | TTATTGTCCAACTACCTGTGGC | GACTTCAAAGTAGCAGCGTCTAT |

**Figure S1 Visualization of propensity score matching**

A Density plot of propensity score before matching.

B Density plot of propensity score after matching.

C Histogram of standardized differences before matching.

D Histogram of standardized differences after matching.

E Dotplot of standardized mean differences.

F Line plot of individual differences.


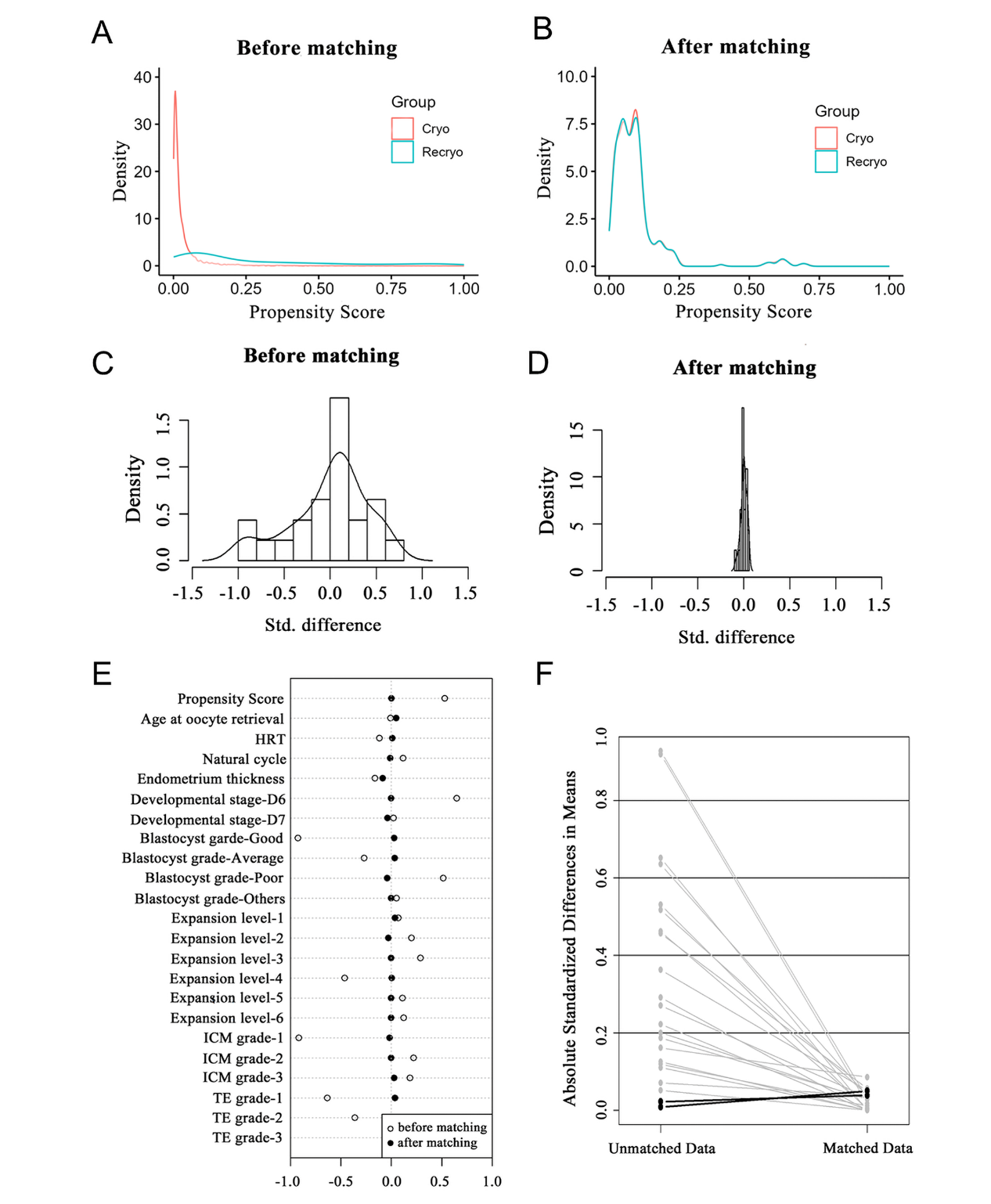

Supplement: Supplementary file 1 — Supporting Information [file MCO2-5-e689-s001.docx]
